# Physiological and molecular response and tolerance of *Macleaya cordata* to lead toxicity

**DOI:** 10.1186/s12864-023-09378-2

**Published:** 2023-05-24

**Authors:** Hongxiao Zhang, Linfeng Hu, Xinlong Du, Xijing Sun, Ting Wang, Zhiying Mu

**Affiliations:** 1 College of Agriculture, Henan University of Science and Technology, Luoyang, 471023 China; 2 College of Biotechnology, Tianjin University of Science and Technology, Tianjin, 300222 China; 3 College of Forestry and Biotechnology, Zhejiang Agriculture and Forestry University, Hangzhou, 311300 China

**Keywords:** *Macleaya cordata*, Transcriptome, Proteome, Pb tolerance, Fe deficiency, Chloroplast metabolism

## Abstract

**Background:**

*Macleaya cordata* is a traditional medicinal herb, and it has high tolerance and accumulation ability to heavy metals, which make it a good candidate species for studying phytoremediation. The objectives of this study were to investigate response and tolerance of *M. cordata* to lead (Pb) toxicity based on comparative analysis of transcriptome and proteome.

**Results:**

In this study, the seedlings of *M. cordata* cultured in Hoagland solution were treated with 100 µmol·L^− 1^ Pb for 1 day (Pb 1d) or 7 days (Pb 7d), subsequently leaves of *M. cordata* were taken for the determination of Pb accumulation and hydrogen peroxide production (H_2_O_2_), meanwhile a total number of 223 significantly differentially expressed genes (DEGs) and 296 differentially expressed proteins (DEPs) were screened between control and Pb treatments. The results showed leaves of *M. cordata* had a special mechanism to maintain Pb at an appropriate level. Firstly, some DEGs were iron (Fe) deficiency-induced transporters, for example, genes of vacuolar iron transporter and three ABC transporter I family numbers were upregulated by Pb, which can maintain Fe homeostasis in cytoplasm or chloroplast. In addition, five genes of calcium (Ca^2+^) binding proteins were downregulated in Pb 1d, which may regulate cytoplasmic Ca^2+^ concentration and H_2_O_2_ signaling pathway. On the other hand, the cysteine synthase upregulated, glutathione S-transferase downregulated and glutathione reductase downregulated in Pb 7d can cause reduced glutathione accumulation and decrease Pb detoxification in leaves. Furthermore, DEPs of eight chlorophyll a/b binding proteins, five ATPases and eight ribosomal proteins can play a pivotal role on chloroplast turnover and ATP metabolism.

**Conclusions:**

Our results suggest that the proteins involved in Fe homeostasis and chloroplast turnover in mesophyll cells may play key roles in tolerance of *M. cordata* to Pb. This study offers some novel insights into Pb tolerance mechanism of plants, and the potential valuable for environmental remediation of this important medicinal plant.

**Supplementary Information:**

The online version contains supplementary material available at 10.1186/s12864-023-09378-2.

## Background

Lead (Pb) is one of the most toxic metals, and Pb in the environment comes from growing industrialization, such as mining activities, increasing vehicles, as well as disposal of sewage, etc. Phytoremediation is a safe and efficient technique, which can remove heavy metals from contaminated soils and ground water with plants that can accumulate large amounts of heavy metals in their bodies [1, 2]. When Pb ions in the soil solution is adsorbed to the root surface, it disturbs uptake of mineral nutrition by roots but also enters passively into transport systems in plants [1]. It was reported that root hairs and secretions can dramatically affect the uptake of Pb by H^+^-ATPase pumps and calcium (Ca) channels [3, 4].

Pb may cause serious damage to living cells even at very low doses by replacing essential elements, such as Ca, iron (Fe) and zinc (Zn), then Pb can disrupt chloroplast and mitochondrion ultrastructure, and cell membrane permeability, and induces the production of reactive oxygen species (ROS), and triggers some enzyme and non-enzymatic antioxidants [4]. ROS include hydrogen peroxide (H_2_O_2_), superoxide anion and hydroxyl radical, and they can oxidize intracellular lipids, proteins, and nucleic acids, thus causing lipid peroxidation, membrane damage, and enzyme inactivation. However, H_2_O_2_ is also involved in regulation of plant metabolism and cellular signaling in response to environmental stresses [4–6].

Plants may prevent the toxic effect of heavy metals by various mechanisms such as adsorption to the cell wall, compartmentation in vacuoles, enhancement of the active efflux, or induction of metal chelates like metallothioneins, phytochelatins, organic (citrates), inorganic (sulphides) complexes and so on [4]. Some plant species can efficiently remove Pb from the soil or water, and they can tolerate high concentrations of Pb far more than ordinary plants, and are regarded as hyperaccumulators of Pb [1, 2]. Studies on the plants for phytoremediation had taken advantage of transcriptomic, proteomic and metabolomic, which analyzed the expression profiles of proteins or genes induced by heavy metals, and found that the plants possess themselves homeostatic mechanism in their tissues or organelles to minimize the damage from varied toxic metals [7, 8].

*Macleaya cordata* (Willd.) is a traditional medicinal herb widely distributed in southern China, and it was also sequenced the first plant genome from the Papaveraceae family [9]. There are various bioactive alkaloids in *M. cordata*, so it was approved by the European Food Safety Authority (EFSA) as a safe plant for the manufacture of feed additives [9]. It was found in tailing areas with high concentration heavy metals, especially soil Pb content reach to 144.6 mg kg^− 1^ and root Pb content reach to 5921 mg kg^− 1^ [10, 11], but had fast growth rate, large biomass and huge taproots. *M. cordata* had been reported as a hyperaccumulator of uranium- or molybdenum-contaminated soil [11, 12]. *M. cordata* also has been found to show good tolerance and accumulation ability to Pb, Zn, cadmium, and manganese [13–16], which make it a good candidate species for studying the phytoremediation. MicroRNAs (miRNAs) related with drought resistant in *Macleaya cordata* had been identified by high-throughput sequencing [17]. In addition, we found that *M. cordata* had very high tolerance to Pb under hydroponic conditions [10], and we also analyze oxidative stress response in roots of *M. cordata* exposed to Pb [16]. On the basis, this research aimed to investigate the tolerance mechanism of *M. cordata* to Pb by comparative transcriptome and proteome.

## Results

### Pb content in roots, stems and leaves of ***Macleaya cordata***

**Fig. 1:**
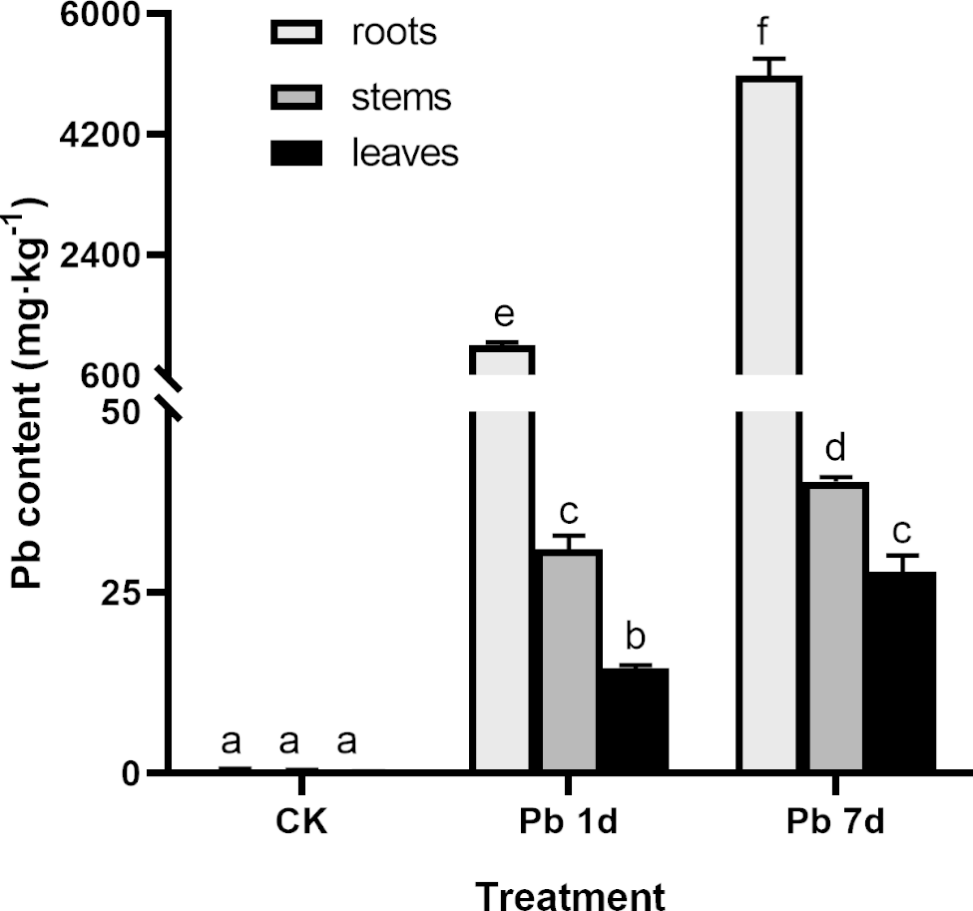
Pb content in roots, stems and leaves of *Macleaya cordata.* Plants were exposed to 0 (CK) or 100 µmol·L^− 1^ Pb 1 day (Pb 1d) or 7 days (Pb 7d). Values are means ± SE (*n* = 3) of three different experiments. Means denoted by letters refer to the significant differences (*p* < 0.05, Duncan’s test)

In the control plants (CK) without Pb treatment, Pb content in roots, stems and leaves are very low, and almost undetectable. After 100 µmol·L^− 1^ Pb treatment for 1 day (Pb 1d) and 7 days (Pb 7d), Pb content in roots had reached respectively 1049 and 5073 mg·kg^− 1^. With the extension of treatment time, there was significantly increase in Pb content of roots, stem and leaves, but the increase of Pb content in stems and leaves was far lower than that in roots (Fig. 1), which showed that high Pb tolerance of *M. cordata* is achieved by limiting Pb transport to shoots.

### H_2_O_2_ determination in leaves of ***Macleaya cordata***

**Fig. 2:**
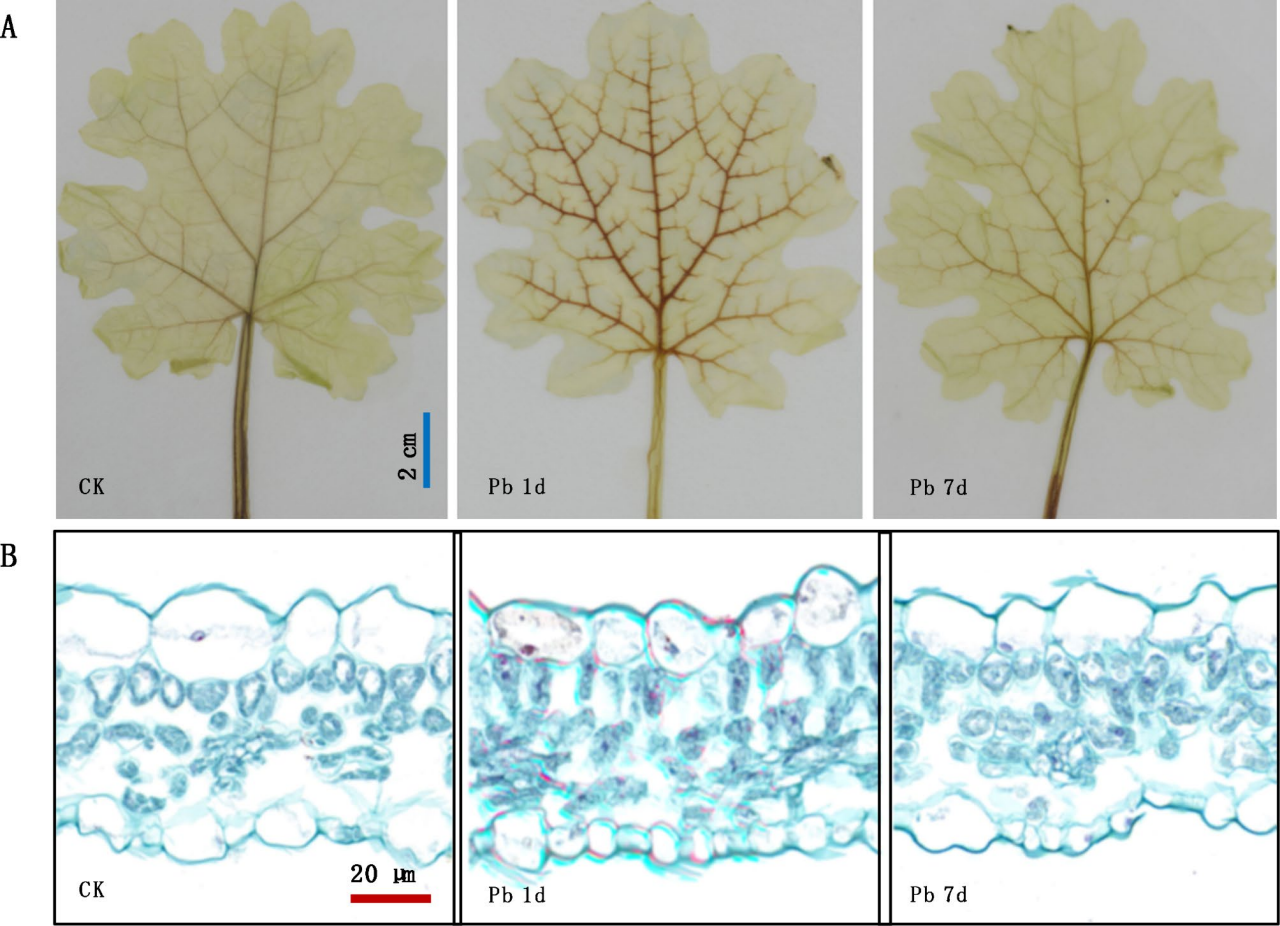
Pb induced H_2_O_2_ production in leaves of *Macleaya cordata.* (A) Histochemical location of H_2_O_2_ by 3,3’-diaminobenzidine staining, bar = 2 cm; (B) Cytochemical location of H_2_O_2_ by paraffin section based on DAB staining, bar = 20 μm. Plants were exposed to control (CK) and 100 µmol·L^− 1^ Pb treatment for 1 day (Pb 1d) or 7 days (Pb 7d). All experiments were repeated at least five times with similar results

The H_2_O_2_ produced was detected by observing the brownish red in leaves of *M. cordata* based on 3,3’-diaminobenzidine (DAB) staining. As shown in Fig. 2A, the veins were lightly stained into brown in leaves of the control and Pb 7d, however, the color of the veins was significantly deepened in Pb 1d. The results of paraffin section showed that a layer of red was detected on the cell walls of mesophyll cells of Pb 1d, and a little red even appeared in intracellular tissue, when compared with that of the controls (Fig. 2B). It hinted that oxidative stress in leaf cells only occurred in the early stage of Pb treatment.

### Comparison transcriptomic and proteomic analysis overview

**Fig. 3:**
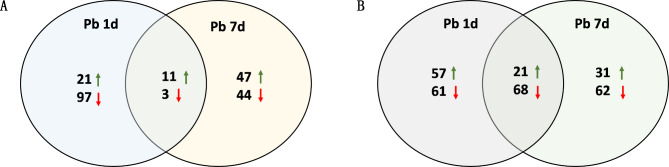
Venn diagrams showing the numbers of differentially expressed genes (A) and proteins (B) in leaves of *Macleaya cordata*. Rising green arrow showed increase and falling red arrow showed decrease in expression of genes or proteins in leaves of *M. cordata* exposed to Pb 1d or Pb 7d, when compared with that of control

32,485 non-redundant transcripts were annotated in the transcriptome of *M. cordata*, and a total number of 223 differentially expressed genes (DEGs) were screened between two sample sets (Pb 1d vs. CK, Pb 7d vs. CK) in present study. An overview of gene expression responses to Pb 1d and Pb 7d is shown in Fig. S1 and Fig. 3A. The results showed that 21 of DEGs were only upregulated in Pb 1d, and 47 were only upregulated in Pb 7d. Furthermore, 97 were only downregulated in Pb 1d, which were almost twice as many as 44 downregulated in Pb 7d. Eleven DEGs upregulated and three DEGs downregulated were found in both Pb 1d and Pb 7d (Fig. 3A). This result showed that Pb 1d induced more DEGs downregulated, and Pb 7d induced more DEGs upregulated in the leaves of *M. cordata*. According to GO annotation classification of genes, extracellular matrix in cellular component and antioxidant activity in molecular function significantly increased under Pb treatment (Fig. S2). Kyoto encyclopedia of genes and genomes (KEGG) pathway analysis (https://www.kegg.jp/kegg/kegg1.html) revealed thatthe enrichment of galactose metabolism increased significantly in leaves of *M. cordata* under Pb 1d, and the enrichment of glyoxylate and dicarboxylate metabolism significantly increased, which followed by glutathione metabolism in leaves of *M. cordata* under Pb 7d (Fig. S3).

After the raw data of proteome were searched against *M. cordata* transcriptome, 296 differentially expressed proteins (DEPs) were screened. The venn diagram of protein expression response to Pb is shown in Fig. 3B. 109 DEPs were identified as upregulated by Pb, most of them upregulated in Pb 1d. 191 DEPs downregulated were identified in this study, Pb 1d and Pb 7d downregulated almost the same number of DEPs. DEPs of 21 upregulated and 68 downregulated were found in both Pb 1d and Pb 7d.

### Characteristics of DEGs involved in Pb tolerance of ***Macleaya cordata***

**Fig. 4:**
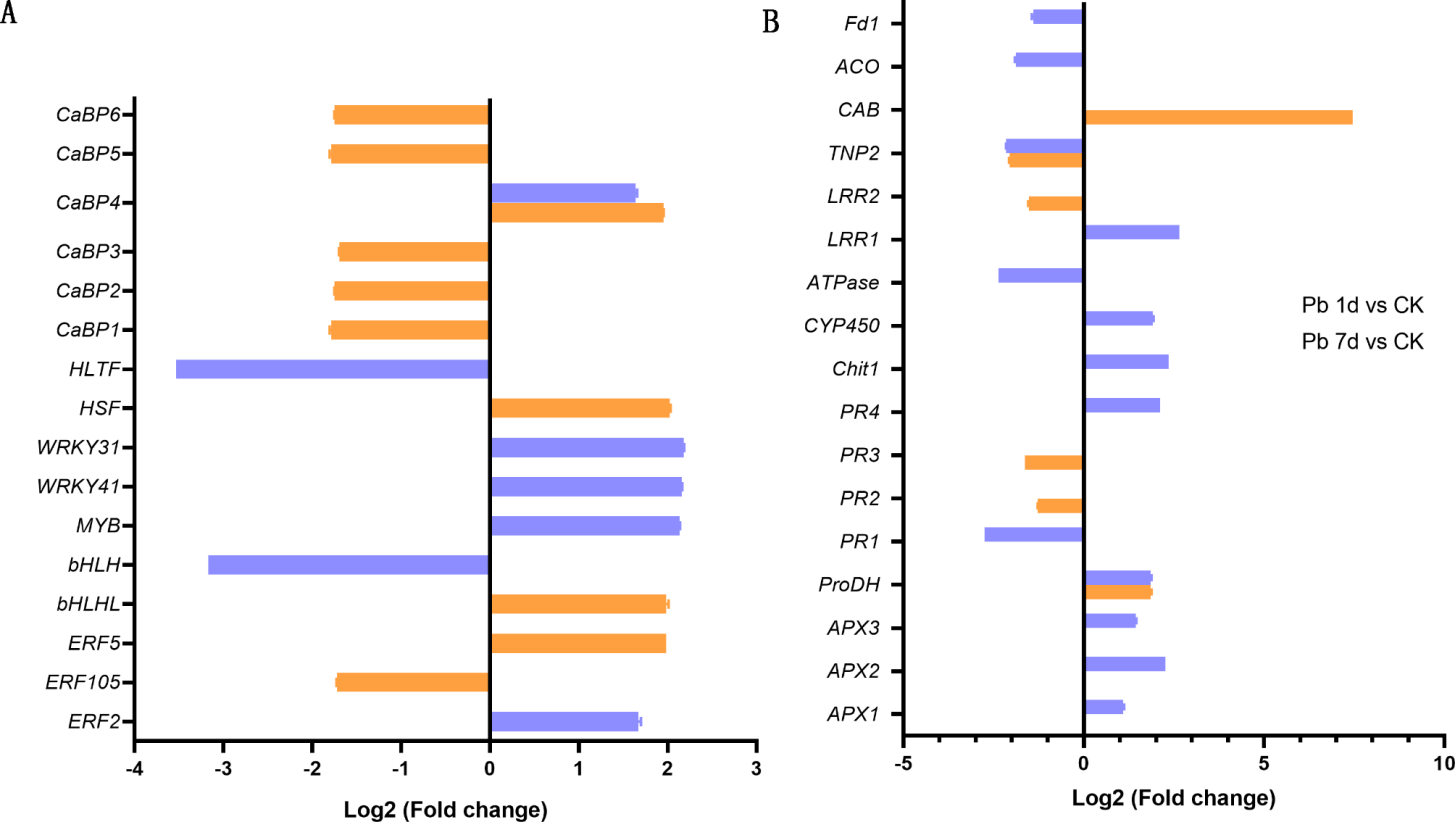
The expression changes of DEGs involved in signal transduction (A) and stress response (B) in leaves of *Macleaya cordata*. Plants were exposed to 0 (CK) or 100 µmol·L^− 1^ Pb 1 day (Pb 1d) or 7 days (Pb 7d). The expression levels of DEGs were showed use Log2 (Fold change) (n = 3, padj < 0.05). ERF: ethylene-responsive transcription factor; bHLH, MYB, WRKY: bHLH, MYB, WRKY transcription factor; HSF: heat stress transcription factor; HLTF: histone-like transcription factor;CaBP: calcium-binding proteins; APX: L-ascorbate peroxidase; ProDH: proline dehydrogenase; PR, pathogenesis-related protein; Chit1: chitinase; CYP450: cytochrome P450; LRR1: leucine rich repeat; TNP2: Transposase; CAB: chlorophyll a-b binding protein; ACO: 1-aminocyclopropane-1-carboxylate oxidase; Fd1: ferredoxin I

Sixteen DEGs were involved in signal transduction pathway (Fig. 4A), and the numbers of DEGs downregulated and upregulated were eight. In downregulated DEGs, five calcium-binding proteins (CaBPs) and one ethylene-responsive transcription factor (ERF) responded to Pb 1d, histone-like (HLTF) and bHLH transcription factor (bHLH) responded to Pb 7d. CaBP4 were upregulated in both Pb 1d and Pb 7d, however, WRKY31, WRKY41, MYB and ERF2 were upregulated in Pb 7d, bHLHL, ERF5 and heat stress transcription factor (HSF) were upregulated in Pb 1d.

Seventeen DEGs were involved in stress response proteins (Fig. 4B), among them, eight were downregulated and nine upregulated by Pb treatment. DEGs of three pathogenesis-related proteins (PR1, PR2 and PR3), ATPase, leucine rich repeat (LRR2), 1-aminocyclopropane-1-carboxylate oxidase (ACO) and ferredoxin I (Fd1) were downregulated in Pb 1d or 7d, only transposase (TNG2) gene was downregulated in both Pb 1d and Pb 7d. On the other hand, the proline dehydrogenase (ProDH) gene was upregulated in both Pb 1d and Pb 7d, and other DEGs, including L-ascorbate peroxidases (APX1, APX2 and APX3), PR4, Chitinase (Chit1), cytochrome P450 (CYP450) and LRR1 were upregulated in Pb 7d, but chlorophyll a-b binding protein (CAB) were significantly upregulated in Pb 1d.

**Fig. 5:**
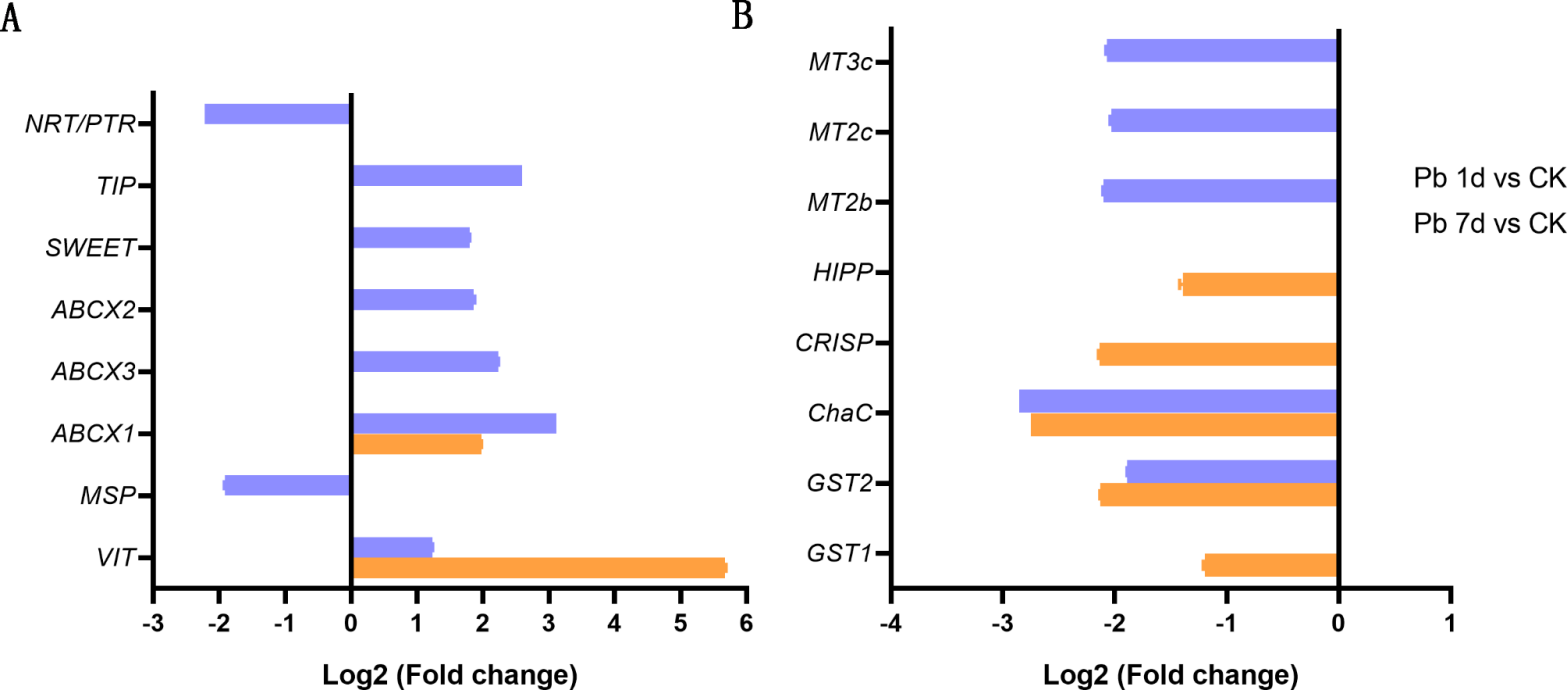
The expression changes of DEGs involved in transporters (A) and cysteine metabolism (B) in leaves of *Macleaya cordata*. Plants were exposed to 0 (CK) or 100 µmol·L^− 1^ Pb 1 day (Pb 1d) or 7 days (Pb 7d). The expression levels of DEGs were showed use Log2 (Fold change) (n = 3, padj < 0.05). VIT, vacuolar iron transporter; MSP: manganese-stabilising protein; ABCX1, ABCX2, ABCX3: ATP-biding cassette (ABC) transporter family members; TIP: aquaporin TIP-type; SWEET: bidirectional sugar transporter; NRT/PTR: nitrate transporter1/peptide transporter. GST: Glutathione S-transferase; ChaC: ChaC-like protein; CRISP: cysteine-rich repeat secretory protein; HIPP: heavy metal-associated isoprenylated plant protein; MTs: metallothionein

Eight transporters, including vacuolar iron transporter (VIT), manganese-stabilizing protein (MSP), three ATP-binding cassette transporter I family members (ABCX1, ABCX2 and ABCX3), bidirectional sugar transporter (SWEET), aquaporin TIP-type (TIP) and nitrate transporter1/peptide transporter family (NRT/PTR) were identified as DEGs in leaves of *M. cordata* to Pb (Fig. 5A). Except *NRT/PTR* and *MSP* were downregulated in Pb 7d, other transporter genes were significantly upregulated in Pb 7d, and *ABCX1* and *VIT* were also upregulated in Pb 1d.

Eight DEGs involved in cysteine metabolism, including glutathione S-transferase (GST1 and GST2), ChaC-like protein (ChaC), cysteine-rich repeat secretory protein (CRISP), heavy metal-associated isoprenylated plant (HIPP) and metallothionein (MT3c, MT2c and MT2b) were downregulated by Pb treatment (Fig. 5B). Among them, three *MTs* responded only to Pb 7d, and *HIPP*, *CRISP* and *GST1* only to Pb 1d, but *ChaC* and *GST2* responded to both Pb 1d and Pb 7d.

### Validation of DEGs by quantitative real-time PCR

**Fig. 6:**
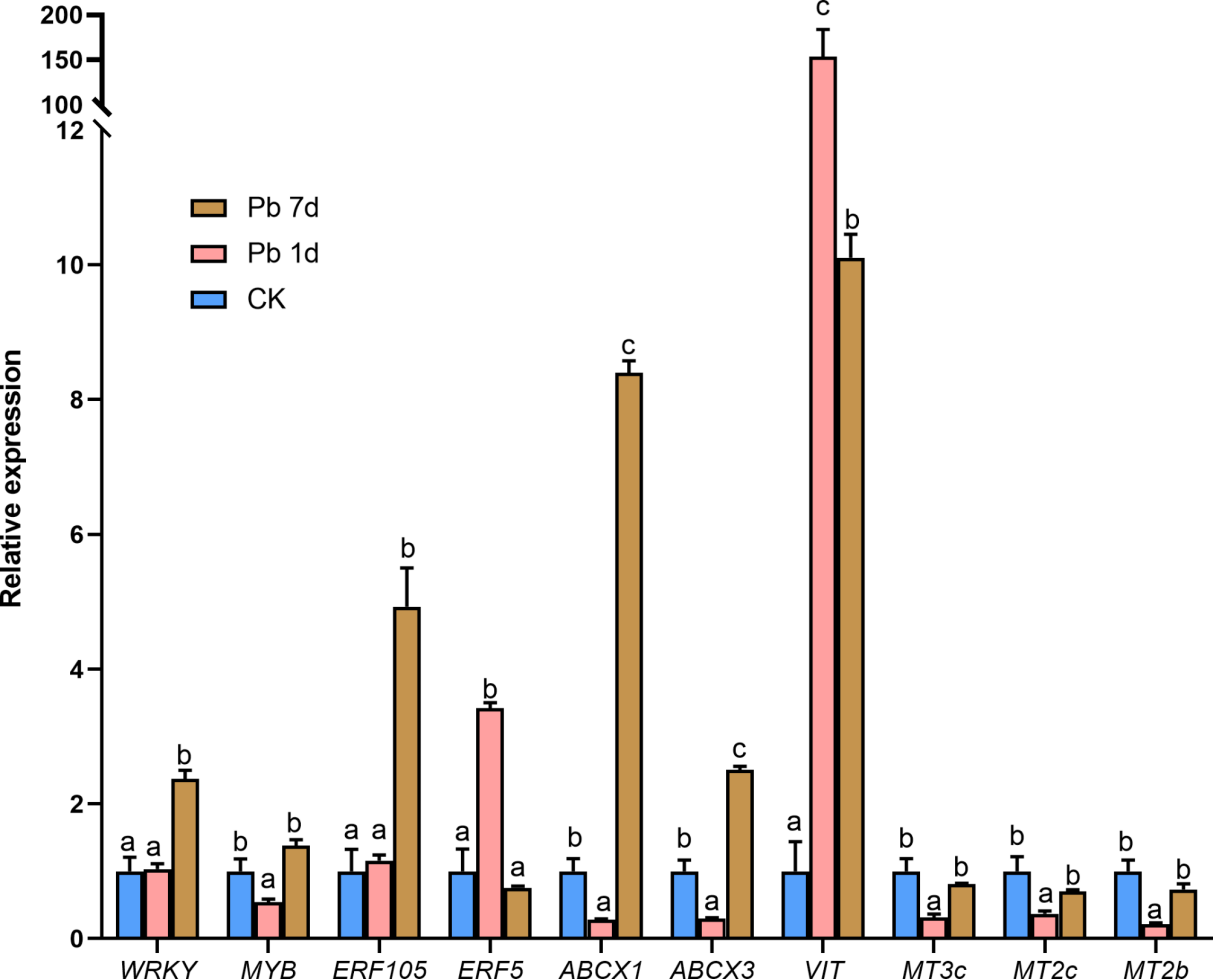
The relative expression of some DEGs in leaves of *Macleaya cordata* by quantitative real-time RCR. Plants were exposed to 0 (CK) or 100 µmol·L^− 1^Pb 1 day (Pb 1d) or 7 days (Pb 7d). The expression levels of DEGs denoted by different letters refers to the significant differences (p < 0.05, Duncan’s test)

According to previous experience, *Mc18s* was selected as the reference gene of *M. cordata* [18]. Ten DEGs were verified the expression change under Pb treatment by quantitative real-time PCR (Fig. 6). The relative gene expression of WRKY41, ERF5 and VIT were entirely consistent with the results of transcriptome; ABCX1 and ABCX3 were consistent with the transcriptome results of Pb 7d; the gene expression of ERF105, MYB, MT3c, MT2c and MT2b were not consistent with the transcriptome results.

### Proteomic profiling of leaves in ***Macleaya cordata*** under Pb treatment

**Fig. 7:**
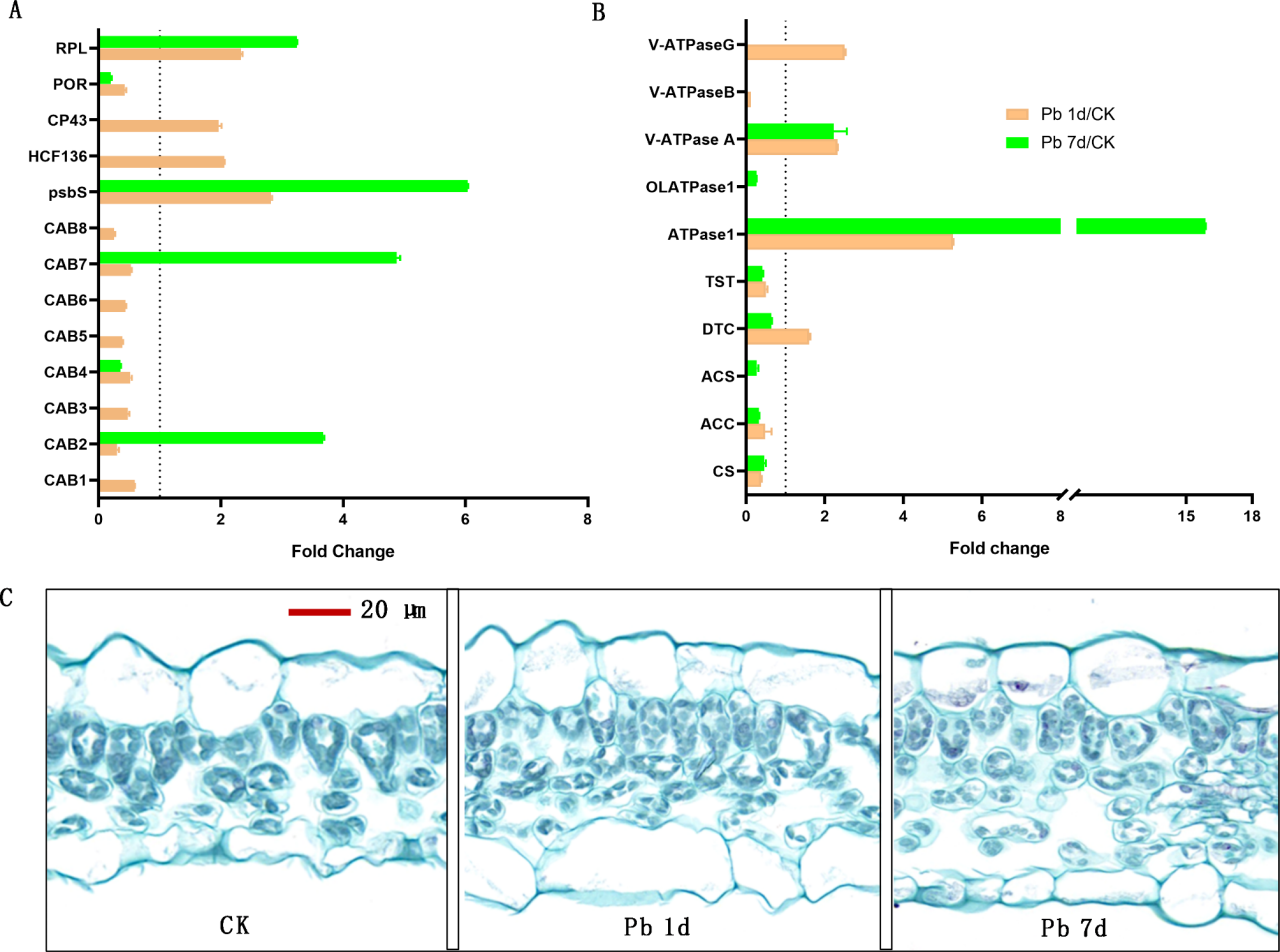
The expression changes of DEPs involved in chlorophyll synthesis (A) and energy metabolism (B), and morphological characteristics of mesophyll cells (C) in leaves of *Macleaya cordata*. Plants were exposed to 0 (CK) or 100 µmol·L^− 1^ Pb 1 day (Pb 1d) or 7 days (Pb 7d). The expression changes of DEPs were showed use Fold Change (*p* < 0.05, Student’s *t*-test). CAB: chlorophyll a/b binding protein; psbS: photosystem II 22 kDa protein; HCF: photosystem II stability/assembly factor; CP43: chlorophyll apoprotein; PRO: protochlorophyllide reductase; RPL: rubisco large subunit-binding protein; CS: citrate synthase; ACC: acetyl-coenzyme A synthetase; ACS: acetyl-CoA carboxylase beta subunit; DTC: mitochondrial dicarboxylate/tricarboxylate transporter; TST: thiosulfate/3-mercaptopyruvate sulfurtransferase; OLATPase: obg-like ATPase; V-ATPase: vacuolar-type ATPase.

As shown in Fig. 7A, eight DEPs were identified as CABs, six of them were downregulated by Pb, and only CAB2 and CAB7 was upregulated in Pb 7d. Moreover, photosystem II 22 kDa protein (psbS), photosystem II stability/assembly factor (HCF), chlorophyll apoprotein (CP43), and rubisco large subunit-binding protein (RPL) were upregulated in Pb 1d, moreover, psbS and RPL were also upregulated in Pb 7d.

Ten DEPs were involved in energy metabolism (Fig. 7B). Two V-type proton ATPases (V-ATPase A and V-ATPase G), ATPase 1 and mitochondrial dicarboxylate/tricarboxylate transporter (DTC) were upregulated in Pb 1d, moreover, V-ATPase A and ATPase 1 were also upregulated in Pb 7d. Other DEPs, including V-ATPase B, obg-like ATPase (OLATPase), thiosulfate/3-mercaptopyruvate sulfurtransferase (TST), acetyl-coenzyme A synthetase (ACS), acetyl-CoA carboxylase beta subunit (ACC) and citrate synthase (CS) were downregulated by Pb. Meanwhile, the degradation of chloroplasts and increase in mesophyll cell numbers was observed in leaves of *M. cordata* after Pb treatment (Fig. 7C).

**Fig. 8:**
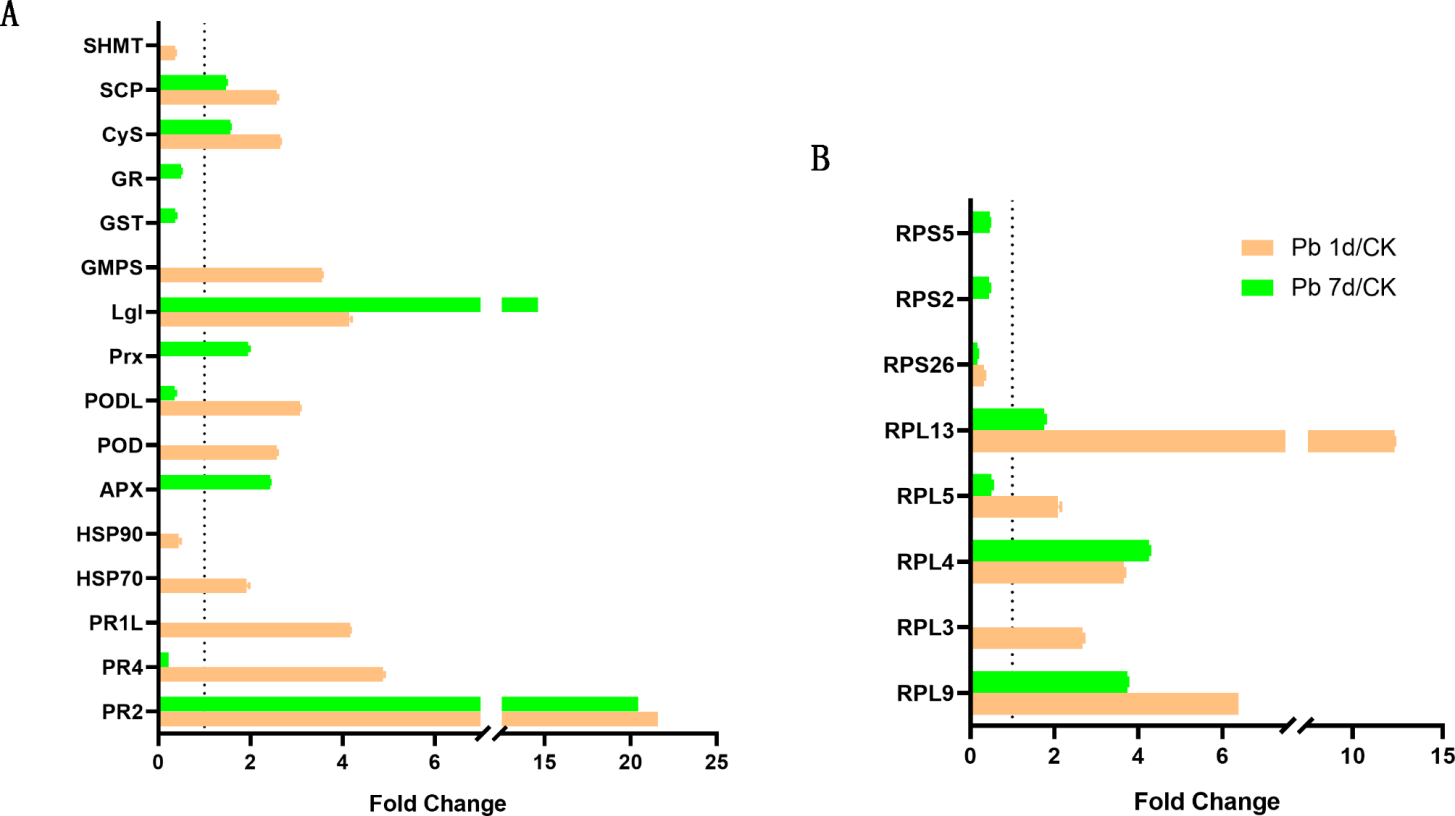
The expression changes of DEPs involved in stress response (A) and ribosomal assembly (B) in leaves of *Macleaya cordata*. Plants were exposed to 0 (CK) or 100 µmol·L^− 1^ Pb 1 day (Pb 1d) or 7 days (Pb 7d). The expression levels of DEPs were showed use Fold Change (*p* < 0.05, Student’s T-test). PRs: pathogenesis-related protein; HSPs: heat shock protein; APX: ascorbate peroxidase; POD: peroxidase; Prx: 2-Cys peroxiredoxin; Lgl: lactoylglutathione lyase; GMPS: GMP synthase; GST: glutathione S-transferase; GR: glutathione reductase; CyS: cysteine synthase; SCP: serine carboxypeptidase; SHMT: serine hydroxymethyltransferase; RPLs: 60 S ribosomal protein; RPSs: 40 S ribosomal protein

As shown in Fig. 8A, sixteen DEPs were involved in stress response. Except for serine hydroxymethyltransferase (SHMT), glutathione reductase (GR), GST, heat shock protein 90 (HSP90), most of DEGs, including three PRs, HSP70, APX, two peroxidases (POD), 2-Cys peroxiredoxin (Prx), lactoylglutathione lyase (Lgl), GMP synthase, cysteine synthase (CyS) and serine carboxypeptidase (SCP) were upregulated by Pb. Eight ribosomal proteins (RPs) were identified by DEPs (Fig. 8B), among them, five 60 S ribosomal proteins (RPL3, RPL4, RPL5, RPL9 and RPL13) were upregulated by Pb, but three 40 S ribosomal proteins (RPS26, RPS2, and RPS5) were downregulated by Pb.

## Discussion

### ***Macleaya cordata*** improves Pb tolerance by restricting Pb transport to shoots

100 µmol·L^− 1^ Pb treatment did not cause a significant change in root elongation, shoot biomass and chlorophyll content of *M. cordata* when compared with the control [10, 16], but Pb contents in roots, stems and leaves of *M. cordata* were increased (Fig. 1). With an extended Pb treatment time, Pb content in leaves was far lower than that in roots, and also significantly lower than that in stems, therefore, *M. cordata* can improve its ability of tolerance by limiting Pb transport to shoots.

### Pb induces H_2_O_2_ production and chloroplast degradation in leaves of ***Macleaya cordata***

H_2_O_2_ production regulates plant tolerance to heavy metal stress as a signal molecular [4]. H_2_O_2_ in leaves induced by Pb is reported in of Arabidopsis [19] and barley [20]. The detected H_2_O_2_ in leaves of *M. cordata* increased significantly under Pb 1d (Fig. 2A). In the paraffin section, the red oxides of H_2_O_2_ were detected in cell walls and chloroplasts of mesophyll cells, but the red stain disappeared under Pb 7d (Fig. 2B), which hinted that H_2_O_2_ in leaves can be a signal molecule to activate the tolerance mechanism of *M. cordata* to Pb.

chloroplast degradation can happen via autophagy, senescence-associated vacuoles and stress-induced chloroplast vesiculation, which has been investigated in Arabidopsis [21–23] and rice [24]. However, it is not the report that chloroplast degradation happened in plants exposed to Pb or other heavy metals treatment. We found the increase amount of mesophyll cells was accompanied by reduction of chloroplasts every cell of *M. cordata* exposed to Pb (Figs. 2B and 7C), which can hint increase of chloroplast turnover under Pb treatment. Sade et al. [24] found chloroplast turnover can increase stress tolerance through the enhancement of nitrogen assimilation in rice. It indicates that the degradation of chloroplasts and the recycling of their nutrients plays a major role in *M. cordata* coping with Pb stress.

### ***Macleaya cordata*** regulates gene or protein expression by Ca^2+^ and H_2_O_2_ signal

Six CaBPs were identified as DEGs of Pb in leaves of *M. cordata*, and five *CaBPs* were downregulated in Pb 1d except *CaBP4* were upregulated in both Pb 1d and Pb 7d (Fig. 4A). It reported that Pb can replace Ca to bind Ca transporters or CaBPs, which causing the decrease of cytoplasmic Ca^2+^ ions [25]. It was reported that one of OsCaBPs enhanced rice tolerance to heat stress by modulating the production of H_2_O_2_ [26]. In addition, ERF2, MYB, WRKY31 and WRKY41 genes were upregulated in Pb 7d (Fig. 4A). *MYB* and *ERF* was reported as DEGs in maize roots in response to Pb stress [27]. It was also reported that overexpression of *ERF* enhances salt, drought, cold and heat tolerance in Arabidopsis [28]. *WRKYs* in *A. thaliana* were upregulated in response to H_2_O_2_ treatment [29], and Cd also upregulated *WRKYs* in Pepper [30].

Plant responses Pb stress by multiple signal pathways, one of them is H_2_O_2_, and cells maintain H_2_O_2_ homeostasis most by antioxidative enzymes [6]. In this study, DEPs of POD, PODL and Prx were upregulated in Pb 1d (Fig. 8A). It was reported that POD had a complex role on H_2_O_2_ production [4], and the activity of POD in plant apoplast and symplast was very different under excess Cu [5] and Cd treatment [6]. Moreover, three APX genes and an APX protein were upregulated in Pb 7d (Figs. 4B and 8A), which can cause a decrease in the production of H_2_O_2_ in leaves of *M. cordata* under Pb 7d (Fig. 2).

Glutathione (GSH) is composed of glutamine, cysteine, and glycine, three essential amino acids, and it is also a strong antioxidant and can bind and transport metal ions to reduce toxicity in cells [31]. Gupta et al. [32] found that GSH played a key role in detoxification of Pb in *Sedum alfredii*. The GSH is oxidized to yield oxidized glutathione (GSSG) by GPX, which is then reduced back to GSH by GR [4]. It was reported that Pb induced producing H_2_O_2_ and decrease of *GST* expression in *Drosophila melanogaster* [33]. In this study, DEGs of *GST2*, DEPs of GST and GR were downregulated in Pb 7d (Figs. 5B and 8A), which can cause GSH accumulation. Meanwhile, cysteine synthase was upregulated by Pb (Fig. 8A), and DEGs dependent on cysteine for protein synthesis, such as *MT2b, MT2c, MT3c, CRISP* and *HIPP* were downregulated by Pb in this study (Fig. 5B), which can increase the synthesis of GSH. Although it has been reported that MTs, CRISP and HIPPs play important roles in metal homeostasis [34]. In addition, two DEPs involved in GSH synthesis, Lgl and GMPS were upregulated by Pb more 4 times than that of control in *M. cordata* (Fig. 8A). Lgl is important for the GSH dependent glutaraldehyde enzyme system in plants [31], and it was upregulated by Pb in this study, which will increase the glyoxylate metabolism and synthesis of GSH. These results show that GSH can be accumulated in leaves of *M. cordata* coping with Pb, which consistent with Pb tolerance mechanism of *S. alfredii* [32].

### ***Macleaya cordata*** decreases Pb toxicity by Fe deficiency signal

Cellular Fe utilization decreases in plants exposed to heavy metal stress [35]. VIT is regarded as an Fe transporter by transporting cytoplasmic Fe ions into vacuoles [36], or maintain Fe homeostasis in plants [37]. In present study of both transcriptome and quantitative real-time PCR result, VIT gene in leaves of *M. cordata* was significantly upregulated by Pb (Figs. 5A and 6), which can transport Fe to vacuolar and lead to Fe deficiency in *M. cordata*. ABC transporter I family numbers are known to play a crucial role in plant secondary metabolites and response to environment stress, including heavy metal stress. In present study, we found three genes of ABC transporter I family numbers, ABCX1, ABCX2 and ABCX3 were upregulated by Pb in transcriptome (Fig. 5A), in addition, genes of ABCX1, ABCX3 and VIT were upregulated by Pb in quantitative real-time RCR (Fig. 6). It was reported that two Arabidopsis *AtABCI10* and *AtABCI11*, were significantly induced by Fe deficiency, regulated chloroplast biogenesis and metal homeostasis [38]. OsABCI7, located on the thylakoid membrane of rice, was reported to regulate the intracellular ROS homeostasis and maintain the stability of thylakoid membrane [39]. AtABCG40 located plasma membrane was regarded as a pump to exclude Pb, including other toxic compounds from the cytoplasm [40, 41]. Therefore, these transporters upregulated in leaves of *M. cordata* may not be responsible for transporting Pb, but rather for improving *M. cordata* tolerance to Pb.

DEGs of TIP and SWEET were also upregulated in Pb 7d (Fig. 5A). Loss of TIP1;1 in Arabidopsis had resulted in early senescence and plant death [42]. The sugars were exported by SWEET transporter family, and plasma membrane-localized AtSWEET13 and AtSWEET14 have also been shown to transport gibberellin [43]. The gibberellin induction of serine carboxypeptidase (SCP) during germination and seeding development has been reported in barley [44] and pea [45]. In present study, SCP was also upregulated in Pb 1d and 7d (Fig. 8A), and it is speculated that the SCP in leaves of *M. cordata* be induced by leaf senescence and gibberellin synthesis.

### ***Macleaya cordata*** maintains Fe homeostasis by chlorophyll and ATP metabolism

Fe is an important component of plant photosynthesis, and Fe deficiency will lead to interveinal chlorosis in developing leaves and a decrease in chlorophyll synthesis [37]. Saito et al. [46] found that Fe deficiency induced CABs expression in barley. Most of CABs except CAB2 and CAB7 were downregulated by Pb in present study, meanwhile, HCF136 and CP43 were upregulated in Pb 1d (Fig. 7A). Chlorophyll affected the stabilization of HCF136 and CP43, then regulated their translation [47]. Moreover, psbS and RPL were significantly upregulated in both Pb 1d and Pb 7d (Fig. 7A). Spinach psbS was also reported as a chlorophyll-binding protein contained a single polypeptide of 22 kDa, and upregulated psbS can enhance photosynthesis in Spinach [48].

Fe deficiency had also induced the downregulation of genes encoding ribosomal proteins in Arabidopsis [49]. Five RPLs upregulated and three RPS downregulated by Pb were identified in leaves of *M. cordata* (Fig. 8B). Mutation of ribosomal proteins in Arabidopsis without exception enhanced leaf variegation, and expression change of ribosomal protein level can affect the balancing of cytosolic and chloroplast translation programs during chloroplast biogenesis [50].

Chlorophyll absorbs light energy and finally provides energy for various biological process of plant, including ion transporter and antioxidant protection. As a result, DEPs involved in ATP metabolism, including DTC, ATPase, V-ATPase A and G were upregulated by Pb treatment in present study (Fig. 7B). It showed that energy synthesis increased under Pb treatment, and it is beneficial to improving *M. cordata* tolerance to Pb. V-ATPases had vital roles in intracellular acidic compartments, has been proven capable of synthesizing ATP in yeast vacuoles [51]. CjDTC in *Citrus junos* was induced by light, salicylic acid and aluminum stress [52], and *PfDTC* in *Plasmodium falciparum* can mediate the oxoglutarate-malate exchange on the inner mitochondrial membrane in order to TCA metabolism [53]. Some key enzymes of TCA pathway, such as ACS, ACC and CS, were downregulated by Pb (Fig. 7B), which may indicate that TCA pathway was partly inhibited in this study. As a result, the degradation of chloroplasts was observed in leaves of *M. cordata* under Pb treatment (Fig. 7C).

## Conclusions

**Fig. 9:**
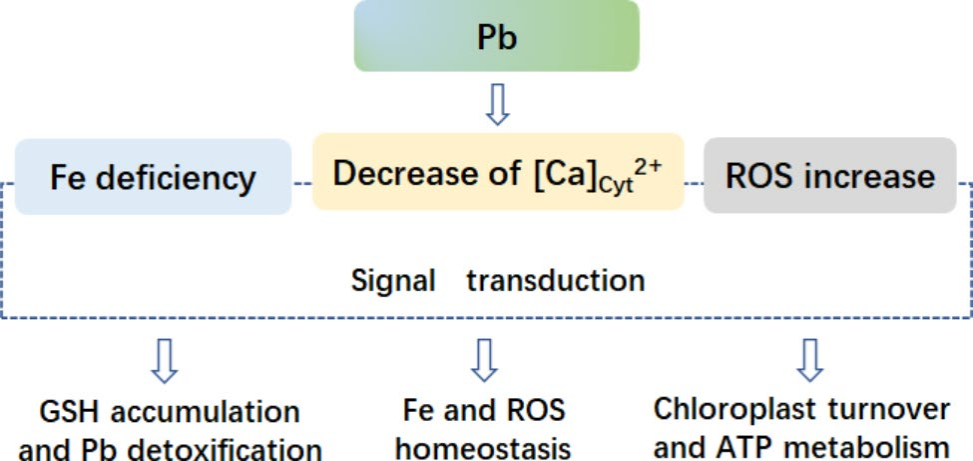
Schematic model of genes and proteins to mediate Pb tolerance mechanism in leaves of *Macleaya cordata*

Based on comparative analysis of transcriptome and proteome of *M. cordata*, it was hypothesized that Pb induce H_2_O_2_ production and Fe deficiency, which subsequently activated a series of expression change of gene or proteins in leaves to cope with Pb stress (Fig. 9). Pb entering cells can replace Ca to bind Ca transporters or CaBPs, which causing the decrease of cytoplasmic Ca^2+^ ions. In addition,Pb caused oxidative stress and Fe deficiency in leaves of *M. cordata*. Intracellular Pb can be combined with GSH to form the compound, which subsequently be transported to vacuoles or apoplasts for reducing toxicity. Furthermore, *VIT* and *ABCXs*, which can be responsible for Fe homeostasis between cytoplasm and chloroplast, and CABs, V-type ATPase and ribosomal proteins can play pivotal role on chloroplast synthesis and ATP metabolism. Our results suggest that the proteins involved in Fe homeostasis and chloroplast turnover in leaf cells may be the keys to Pb tolerance of *M. cordata*.

## Methods

### Plant material and hydroponic experiment

The seeds of *M. cordata* were collected from the tailing of Huaguoshan town of Luoyang city in China (Lat. 39°19’ N, Long. 111°53’ E). After the seeds were germinated in vermiculite, and then, seedlings were cultured with Hoagland solution under the same conditions as in Zhang et al. [10]. The solution pH was adjusted to 5.3 with renewal of the nutrient solution every 2 days. Uniform seedlings with four leaves were treated with 0 (CK) or 100 µmol·L^− 1^ Pb(NO_3_)_2_ in a complete Hoagland solution for 1 day (Pb 1d) or 7 days (Pb 7d). Three biological repeats were set for each treatment. After exposure, roots, stems and leaves of *M. cordata* were collected for Pb content, the second youngest leaves were separated for analysis of transcriptome and proteome, quantitative real-time RCR and detection of H_2_O_2_in situ.

### Determination of Pb content

Roots, stems and leaves of *M. cordata* were collected, washed, dried and digested following the procedure described by Zhang et al. [10]. The ICP-OES (Optima 8000, PerkinElmer, USA) was used to analyze the contents of Pb.

### Histochemical detection of H_2_O_2_

H_2_O_2_ was detected by the DAB method as described by Zhang et al. [54]. The second youngest leaves of *M. cordata* were cut and immersed in DAB solution (1 mg·mL^− 1^, pH 3.8), vacuum-infiltrated, incubated, bleached, and photographed as before [54].

### Morphological observation of mesophyll cells

The leaves from DAB staining or without staining were cut into small pieces, and immersed in FAA fixative solution (Gefan Biotech, Shanghai) for more than 24 h, and dehydrated through an ethanol dehydration series at room temperature. Subsequently, samples were embedded in paraffin blocks and 15 μm-thickness sections were prepared according to the method of Maniou et al. [55]. Sections were stained following Johansen’s safranin and fast green protocol [56]. Under the microscope, if red appears in the green cells compared with the control, indicating that H_2_O_2_ is produced in this area.

### Transcriptome sequencing

Total RNA was extracted using a FastPure Plant Total RNA Isolation kit (Vazyme, Nanjing, China) according to the manufacturer’s instructions. After passing the library inspection, high-throughput sequencing was then performed using the HiSeq 2000 sequencing platform at Genepioneer Biotech (Nanjing, China) according to the method of Guan et al. [57]. 104.02 Gb clean data were obtained after raw data were filtered, and the information of sequencing data was shown Table S1. The sequenced reads were assembled with Trinity software to obtain 116,944 transcripts and 58,583 unigenes. The transcript sequences were compared with genome sequence of *M. cordata* to assemble the cds unigenes (www.ncbi.nlm.nih.gov/nuccore/MVGT01004176), and annotations of unigenes were obtained using the NCBI (nr), Swiss-port, GO, COG, KOG and KEGG databases [9, 58]. The Benjamini-Hochberg correction method is adopted to adjust the significance *p* value (padj), to control the proportion of false positives in the final analysis results. A padj < 0.05 and |log2 (fold change) |≥ 1 were set as the thresholds for significant differential expression between Pb treatments and the control.

### Quantitative real-time RCR

RNA was extracted using a FastPure Plant Total RNA Isolation kit (Vazyme, Nanjing, China), and reverse transcribed using Hifair™ II 1st Strand cDNA Synthesis for quantitative real-time PCR (Yeasen, Shanghai, China) according to the manufacturer’s instructions. The primers were designed online (https://sg.idtdna.com/PrimerQuest/Home/Index) according the sequences of unigenes from *M. cordata* transcriptome (Table S2). Bio-Rad CFX System and Hifair III One Step quantitative real-time PCR SYBR Green Kit (Yeasen, Shanghai, China) were used for quantitative real-time PCR analysis. The specificity of amplified PCR products was verified by melting curve analysis, and all tested unigenes were normalized to the reference gene *Mc18s*.

### Proteome analysis

The proteins were extracted in acetone, and the protein concentration was determined using a modified Bradford Protein Assay Kit according to the manufacturer’s instructions. About 500 µg proteins were resuspended in 50 mM Tris-HCl (pH 8.0) with 8 M urea and 1 M DTT for proteomic analysis. Subsequently, protein was digested with trypsin (Promega) and the peptide products (1.5 µg) were separated by reverse-phase liquid chromatography (DIONEX Thermo Scientific). Proteomic analysis was performed using tandem mass spectrometry (Thermo Scientific, LTQ-Orbitrap) based label-free quantification according to the method of Duan et al. [59]. The resulting MS/MS raw data were searched against *M. cordata* transcriptome database using MaxQuant 2.1 software (Max Planck Institute of Biochemistry) with the searching parameters as Duan et al. [59]. A 1.5-fold change (up)/0.67-fold change (down) change and *t*-test (*p* < 0.05) as the minimum cut off between Pb treatments and the control. Related information of DEPs in *M. cordata* leaves under Pb treatment was shown in Table S3.

### Statistical analysis

Data were analyzed using IBM SPSS Statistics 25 and drew with GraphPad Prism 9. All of data are the mean value ± SE of three independent replicates; means denoted by different letters refer to the significant differences (*p* < 0.05, Duncan’s test). Experiments of staining were repeated at least five times with similar results.

## Electronic supplementary material

Below is the link to the electronic supplementary material.

Supplementary Material 1

Supplementary Material 2

## Data Availability

The data and materials supporting the conclusions of this study are included within the article and its additional files. The raw datasets generated during the current study are available in ProteomeXchange Consortium (http://proteomecentral.proteomexchange.org) via the iProX partner repository with the project PXD038143.

## Declarations

### Rights and permissions

The seeds of *M. cordata* are stored in the laboratory “Resource Plant Evaluation and Innovative Utilization”, College of Agriculture, Henan University of Science and Technology, which has been used in our published article (Reference 10). All methods in this manuscript were carried out in accordance with the guidelines presented with relevant references, and all results had been repeated according to these methods.

## Abbreviations

DAB: 3,3’-diaminobenzidine
H_2_O_2_: hydrogen peroxide
DEG: differentially expressed gene
DEP: differentially expressed protein
Pb: lead
Fe: iron
Ca: calcium
VIT: vacuolar iron transporter
ABC: ATP-biding cassette (ABC) transporter.
