# Supplementary material for: Physiological and molecular response and tolerance of Macleaya cordata to lead toxicity
Source: BMC Genomics. 2023 May 24;24:277. doi: 10.1186/s12864-023-09378-2 (PMC10210377; doi:10.1186/s12864-023-09378-2)
Supplement: Supplementary file 1 — Supplementary Material 1 [file 12864_2023_9378_MOESM1_ESM.docx]

**Table S1** Transcriptome sequencing data of *Macleaya cordata* leaves under Pb treatment.

| Samples | Read Number | Base Number | GC Content | %≥Q30 |
| --- | --- | --- | --- | --- |
| CK-1 | 24079770 | 7223931000 | 43.52 | 92.78 |
| CK-2 | 22351713 | 6705513900 | 44.08 | 93.72 |
| CK-3 | 18952705 | 5685811500 | 43.34 | 95.08 |
| Pb 1d-1 | 21230098 | 6369029400 | 44.73 | 95.02 |
| Pb 1d-2 | 25200398 | 7560119400 | 44.09 | 92.17 |
| Pb 1d-3 | 22563960 | 6769188000 | 43.74 | 92.43 |
| Pb 7d-1 | 24929413 | 7478823900 | 42.24 | 91.68 |
| Pb 7d-2 | 26437092 | 7931127600 | 44.20 | 95.20 |
| Pb 7d-3 | 27233886 | 8170165800 | 44.46 | 95.00 |

**Table S2** Primers of some genes for quantitative real-time PCR.

| **Primer name** | **Sequence (from 5’ to 3’)** |
| --- | --- |
| Mc18S-F | CTTCGGGATCGGAGTAATGA |
| Mc18S-R | GCGGAGTCCTAGAAGCAACA |
| McWRKY-F | TGGAGAAAGCCCTGAATTGG |
| McWRKY-R | AAGATGAAGACGGGTTGAGATG |
| McMYB-F | GGAATCTGACCCACCAACAA |
| McMYB-R | AACCCAGCAGGAACATTCTC |
| McERF105-F | TGGACGTCTATTTGGGAGAATG |
| McERF105-R | GATGACCATCAGCTGGGAATAA |
| McERF5-F | TTATGATCGTGCGGCGTATAA |
| McERF5-R | AAGCCTCGAGTTGTCTCTAAAC |
| McABCX1-F | CGGTCGCAGACAACATAAGA |
| McABCX1-R | AAGGTCAGCAAGGTTGAGTAG |
| McABCX3-F | AGTGGCAGTGGCAAATCA |
| McABCX3-R | AGGGAGAGAACATCGAGATCA |
| McVIT-F | GGTCTCCACAGCATCTTTGA |
| McVIT-R | ACTACAAGCTCCGGCTACTA |
| McMT2c-F | TGTGGAGGATGTGGGATGTA |
| McMT2c-R | CTCCCTCAACCTTTCCATAGTTC |
| McMT3c-F | TGTTCTTCTGCTCCAACGTC |
| McMT3c-R | GTGAGGAAGGAGAGCCAATATG |
| McMT2b-F | CTGTGGAGGATGCAAGATGTA |
| McMT2b-R | CCGAAGCTTGTCTTCTTAGGT |

**Table S3** Information of differentially expressed proteins in *Macleaya cordata* leaves under Pb treatment.

| Unigenes IDs | Annotation | Protein names | Unique peptides | Sequence coverage [%] | MW  [kDa] | Score |
| --- | --- | --- | --- | --- | --- | --- |
| TRINITY_DN11357_c1_g3 | chlorophyll a-b binding protein 8 | CAB1 | 4 | 23.0 | 30.3 | 56.7 |
| TRINITY_DN16770_c1_g1 | chloroplast light harvesting chlorophyll A/B binding protein | CAB2 | 1 | 28.1 | 28.3 | 87.2 |
| TRINITY_DN16870_c0_g2 | chlorophyll a-b binding protein 4 | CAB3 | 3 | 16.7 | 27.8 | 41.7 |
| TRINITY_DN18310_c1_g2 | chlorophyll a-b binding protein CP26 | CAB4 | 7 | 33.9 | 33.0 | 264.3 |
| TRINITY_DN23689_c1_g2 | chlorophyll a-b binding protein of LHCII type 1 | CAB5 | 2 | 19.8 | 25.3 | 32.1 |
| TRINITY_DN23689_c1_g5 | chlorophyll a-b binding protein of LHCII type 1 | CAB6 | 1 | 31.9 | 17.0 | 51.3 |
| TRINITY_DN24022_c0_g4 | chlorophyll a-b binding protein | CAB7 | 2 | 10.2 | 29.4 | 24.1 |
| TRINITY_DN35913_c0_g1 | chlorophyll a-b binding protein 13 | CAB8 | 2 | 17.8 | 16.5 | 39.1 |
| TRINITY_DN22033_c0_g1 | photosystem II 22 kDa protein | psbS | 3 | 22.6 | 21.0 | 21.2 |
| TRINITY_DN24651_c0_g2 | photosystem II stability/assembly factor HCF136 | HCF136 | 5 | 15.9 | 46.4 | 323.3 |
| TRINITY_DN29365_c0_g1 | photosystem II CP43 chlorophyll apoprotein, partial | CP43 | 5 | 17.1 | 51.8 | 258.2 |
| TRINITY_DN23108_c0_g1 | protochlorophyllide reductase | POR | 1 | 9.8 | 27.2 | 7.0 |
| TRINITY_DN21093_c0_g1 | ruBisCO large subunit-binding protein subunit beta | RPL | 4 | 9.7 | 63.8 | 41.5 |
| TRINITY_DN20910_c0_g1 | citrate synthase, mitochondrial | CS | 1 | 4.0 | 52.6 | 12.7 |
| TRINITY_DN26093_c1_g2 | acetyl-CoA carboxylase beta subunit (chloroplast) | ACC | 2 | 4.1 | 56.0 | 19.2 |
| TRINITY_DN28807_c2_g1 | acetyl-coenzyme A synthetase, chloroplastic/glyoxysomal-like isoform X1 | ACS | 1 | 2.1 | 79.2 | 11.2 |
| TRINITY_DN28763_c0_g1 | mitochondrial dicarboxylate/tricarboxylate transporter DTC | DTC | 2 | 10.1 | 24.2 | 41.7 |
| TRINITY_DN21470_c0_g1 | thiosulfate/3-mercaptopyruvate sulfurtransferase 1, mitochondrial | TST | 1 | 3.1 | 41.5 | 19.2 |
| TRINITY_DN28936_c0_g6 | ATPase subunit 1 | ATPase1 | 5 | 13.6 | 55.4 | 160.1 |
| TRINITY_DN15303_c0_g1 | obg-like ATPase 1 isoform X1 | OLATPase1 | 2 | 5.6 | 40.2 | 14.6 |
| TRINITY_DN29420_c3_g1 | V-type proton ATPase catalytic subunit A [Nelumbo nucifera] | V-ATPase A | 4 | 10.4 | 62.5 | 323.3 |
| TRINITY_DN6247_c0_g2 | V-type proton ATPase subunit B3-like | V-ATPase B | 1 | 13.0 | 18.0 | 15.2 |
| TRINITY_DN24239_c0_g1 | V-type proton ATPase subunit G1-like isoform X1 | V-ATPase G | 2 | 30.9 | 12.3 | 144.7 |
| TRINITY_DN11394_c0_g1 | pathogenesis-related protein STH-2-like | PR2 | 1 | 9.1 | 17.7 | 21.5 |
| TRINITY_DN27153_c0_g1 | pathogenesis-related protein PR-4 | PR4 | 3 | 36.6 | 15.7 | 240.3 |
| TRINITY_DN26434_c1_g1 | basic form of pathogenesis-related protein 1-like | PR1L | 6 | 62.4 | 18.3 | 323.3 |
| TRINITY_DN17103_c0_g1 | heat shock 70 kDa protein 15-like | HSP70 | 3 | 4.8 | 93.6 | 18.3 |
| TRINITY_DN30609_c0_g1 | heat shock protein 90-1-like | HSP90 | 1 | 14.9 | 11.0 | 11.6 |
| TRINITY_DN26300_c0_g1 | L-ascorbate peroxidase 2, cytosolic-like | APX | 3 | 44.2 | 13.2 | 231.3 |
| TRINITY_DN26172_c0_g1 | peroxidase 2b | POD | 7 | 25.1 | 37.1 | 323.3 |
| TRINITY_DN27396_c1_g4 | peroxidase 51-like | PODL | 2 | 7.1 | 42.0 | 17.0 |
| TRINITY_DN29437_c1_g4 | 2-Cys peroxiredoxin BAS1, chloroplastic-like | Prx | 1 | 11.7 | 22.8 | 12.7 |
| TRINITY_DN23240_c0_g1 | lactoylglutathione lyase, chloroplast | Lgl | 2 | 8.0 | 41.5 | 19.3 |
| TRINITY_DN21320_c0_g1 | GMP synthase | GMPS | 1 | 2.8 | 59.6 | 7.9 |
| TRINITY_DN23466_c0_g1 | glutathione S-transferase U10-like | GST | 1 | 9.7 | 14.4 | 27.6 |
| TRINITY_DN28878_c0_g1 | glutathione reductase, chloroplastic-like | GR | 1 | 2.7 | 60.3 | 8.6 |
| TRINITY_DN24664_c0_g1 | cysteine synthase | CS | 3 | 12.5 | 31.9 | 28.5 |
| TRINITY_DN26298_c0_g1 | serine carboxypeptidase-like 35 | SCP | 2 | 7.5 | 54.6 | 12.9 |
| TRINITY_DN24531_c0_g1 | serine hydroxy-methyltransferase | SHMT | 2 | 6.4 | 39.6 | 288.7 |
| TRINITY_DN11011_c0_g1 | 60S ribosomal protein L9 | RPL9 | 1 | 14.0 | 12.1 | 6.2 |
| TRINITY_DN16232_c0_g1 | 60S ribosomal protein L3 | RPL3 | 1 | 6.3 | 27.3 | 146.9 |
| TRINITY_DN19016_c0_g1 | 60S ribosomal protein L4 | RPL4 | 1 | 10.4 | 44.8 | 22.4 |
| TRINITY_DN27903_c1_g3 | 60S ribosomal protein L5 | RPL5 | 2 | 5.3 | 34.8 | 50.2 |
| TRINITY_DN22722_c0_g2 | 60S ribosomal protein L26-1 | RPL13 | 1 | 8.2 | 16.7 | 14.1 |
| TRINITY_DN21052_c0_g1 | 30S ribosomal protein 2, chloroplastic | RPS26 | 1 | 7.9 | 22.8 | 18.8 |
| TRINITY_DN25756_c0_g1 | 40S ribosomal protein S2-2-like | RPS2 | 2 | 14.1 | 20.0 | 21.6 |
| TRINITY_DN18887_c0_g1 | 40S ribosomal protein S5-like | RPS5 | 1 | 7.2 | 20.0 | 36.4 |
| TRINITY_DN11011_c0_g1 | 60S ribosomal protein L9-1 | RPL9 | 1 | 14.0 | 12.1 | 6.2 |
