# Supplementary material for: Physiological and molecular response and tolerance of Macleaya cordata to lead toxicity
Source: BMC Genomics. 2023 May 24;24:277. doi: 10.1186/s12864-023-09378-2 (PMC10210377; doi:10.1186/s12864-023-09378-2)
Supplement: Supplementary file 2 — Supplementary Material 2 [file 12864_2023_9378_MOESM2_ESM.docx]

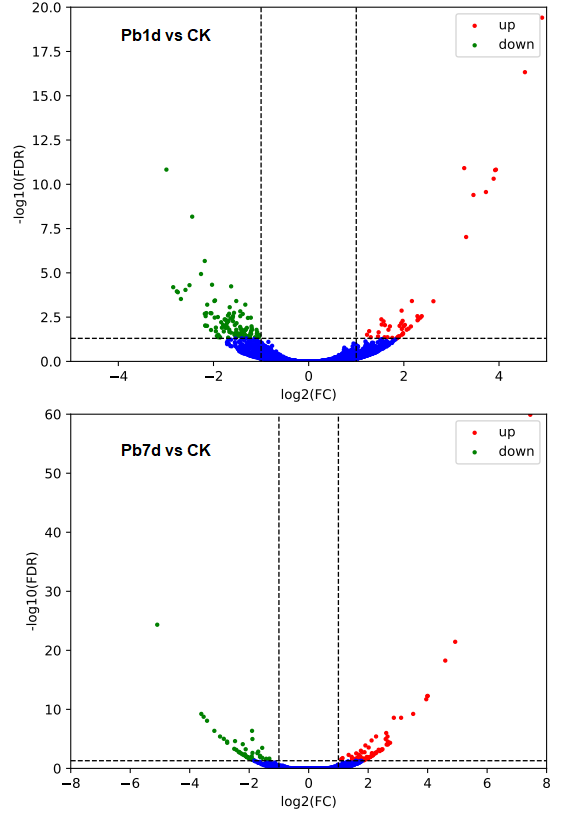


**Figure S1** Volcano diagram of differentially expressed genes in *Macleaya cordata* leaves under Pb treatment.


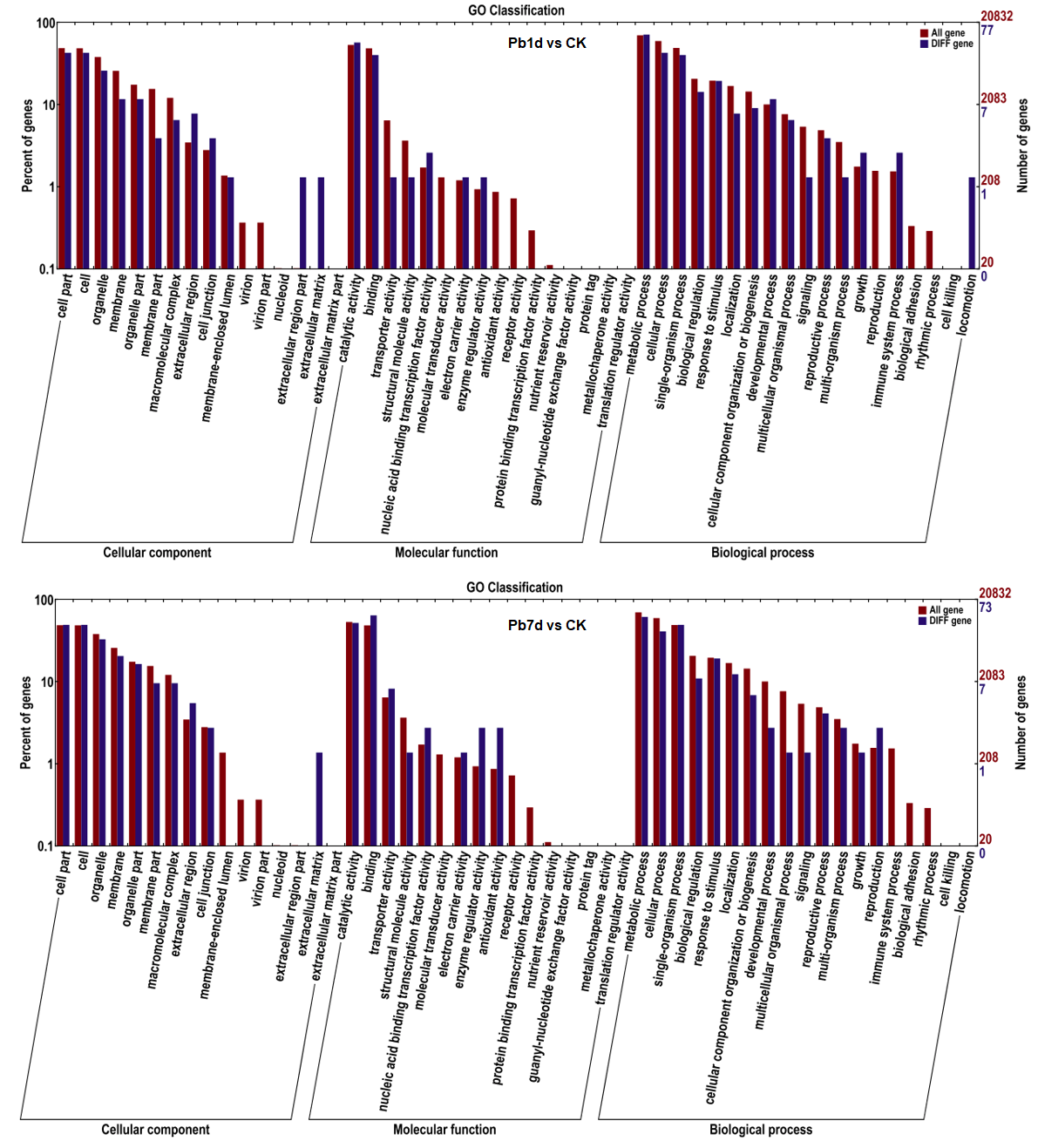


**Figure S2** GO annotation classification of all genes and differentially expressed genes (DIFF) in *Macleaya cordata* leaves under Pb treatment.


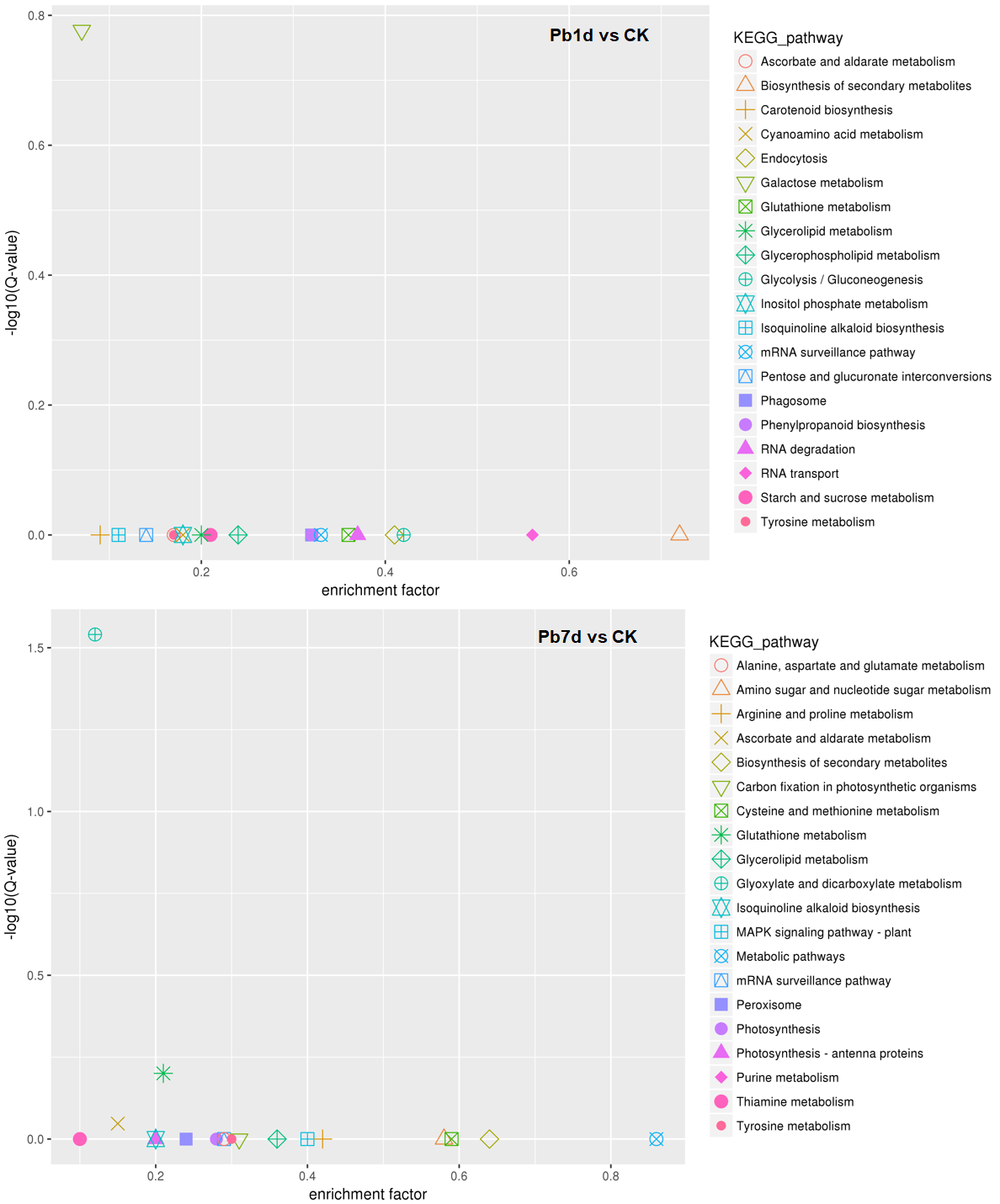


**Figure S3** KEGG pathway enrichment of differentially expressed genes in *Macleaya cordata* leaves under Pb treatment. The smaller the enrichment factor, the more significant the enrichment level of differentially expressed genes in this pathway. The larger the ordinate is, the more reliable the enrichment significance of differentially expressed genes in this pathway is.
